# Identification of a Compound That Inhibits the Growth of Gram-Negative Bacteria by Blocking BamA–BamD Interaction

**DOI:** 10.3389/fmicb.2020.01252

**Published:** 2020-06-19

**Authors:** Yan Li, Xiaohong Zhu, Jing Zhang, Yuan Lin, Xuefu You, Minghua Chen, Yanchang Wang, Ningyu Zhu, Shuyi Si

**Affiliations:** ^1^Beijing Key Laboratory of Antimicrobial Agents, Institute of Medicinal Biotechnology, Chinese Academy of Medical Sciences and Peking Union Medical College, Beijing, China; ^2^State Key Laboratory of Bioactive Substance and Function of Natural Medicines, Institute of Materia Medica, Chinese Academy of Medical Sciences, Beijing, China; ^3^Department of Biomedical Sciences, College of Medicine, Florida State University, Tallahassee, FL, United States

**Keywords:** outer-membrane proteins, *Escherichia coli*, BamA–BamD, yeast two-hybrid, antibacterial agent

## Abstract

The demand for novel antibiotics is imperative for drug-resistant Gram-negative bacteria which causes diverse intractable infection disease in clinic. Here, a comprehensive screening was implemented to identify potential agents that disrupt the assembly of β-barrel outer-membrane proteins (OMPs) in the outer membrane (OM) of Gram-negative bacteria. The assembly of OMPs requires ubiquitous β-barrel assembly machinery (BAM). Among the five protein subunits in BAM, the interaction between BamA and BamD is essential for the function of this complex. We first established a yeast two-hybrid (Y2H) system to confirm the interaction between BamA and BamD, and then screened agents that specifically disrupt this interaction. From this screen, we identified a compound IMB-H4 that specially blocks BamA–BamD interaction and selectively inhibits the growth of *Escherichia coli* and other Gram-negative bacteria. Moreover, our results suggest that IMB-H4 disrupts BamA–BamD interaction by binding to BamA. Strikingly, *E. coli* cells having been treated with IMB-H4 showed impaired OM integrity and decreased the abundance of OMPs. Therefore, an antibacterial agent was identified successfully using Y2H system, and this compound likely blocks the assembly of OMPs by targeting BamA–BamD interaction in Gram-negative bacteria.

## Introduction

Global health has been subjected in jeopardy as a result of emerging strains of drug-resistant Gram-negative bacteria (Wellington et al., 2013). However, very limited drugs are available for Gram-negative bacteria infection treatment (Lepore et al., 2019). The major hurdle for efficient elimination of infection is the presence of outer membrane (OM) in Gram-negative bacteria. The OM is a double-layer hydrophobic structure that envelopes the bacteria and functions as a highly selective permeability barrier, which facilitate bacteria with resistance to unfavorable surrounding environment and antibiotics, thereby deactivating many antibiotics prescriptions in the clinic (Nikaido, 2003). Antibiotic targeting the OM structure would have potential to kill Gram-negative bacteria or sensitize them to antibiotics unable to penetrate through the OM.

The OM is composed of lipid bilayer with integral β-barrel OM proteins (OMPs) (Nikaido, 2003; Silhavy et al., 2010). OMPs are critical for OM to exercise its functions; meanwhile, it is noteworthy that OMPs are frequently associated with bacterial virulence (Henderson et al., 1998; Leo et al., 2012). Therefore, disrupting the proper assembly and folding of OMPs would likely to impair OM integrity and inhibit the growth of Gram-negative bacteria.

The assembly of OMPs in OM requires a multi-protein complex known as β-barrel assembly machinery (BAM) (Wu et al., 2005; Ricci and Silhavy, 2012). The BAM complex consists of multiple components which can vary among species (Volokhina et al., 2009; Anwari et al., 2010, 2012; Paramasivam et al., 2012; Webb et al., 2012; Noinaj et al., 2013). BamA is the core component of BAM complex to perform protein transport/assembly functions, and this protein is highly conserved in Gram-negative bacteria and essential for cell viability (Bos and Tommassen, 2004; Voulhoux and Tommassen, 2004; Wu et al., 2005; Knowles et al., 2009; Anwari et al., 2012). In addition to BamA, the BAM complex, at least in *Escherichia coli*, includes four lipoproteins BamB, BamC, BamD, and BamE. Among them, only BamD is essential and conserved in most Gram-negative bacteria (Malinverni et al., 2006; Kim et al., 2007; Sklar et al., 2007; Hagan et al., 2010; Anwari et al., 2012). BamB and BamE are mainly present in α-, β-, and γ-proteobacteria, while BamC is present only in β- and γ-proteobacteria. A new lipoprotein subunit, named BamF, is present exclusively in α-proteobacteria (Anwari et al., 2012). BamA contains a C-terminal transmembrane β-barrel domain, and substrate proteins can be integrated into OM by laterally passage along the lumen of BamA β-barrel (Gatzeva-Topalova et al., 2008). In addition to the β-barrel domain, BamA also incorporates a large N-terminal periplasmic domain, which consists of Polypeptide-Transport-Associated (POTRA) repeats (Arnold et al., 2010; Koenig et al., 2010). The number of POTRA domain varies in BamA proteins from different bacterial species, but BamA proteins from majority Gram-negative bacteria, including *E. coli*, have five POTRA domains (Arnold et al., 2010; Koenig et al., 2010). The POTRA domains are required to recruit other components of the BAM complex, such as BamB–E in *E. coli* (Voulhoux et al., 2003; Gatzeva-Topalova et al., 2010; Jansen et al., 2015; Bergal et al., 2016; Fleming et al., 2016). The N-terminal domain of BamD interacts with OMP substrates to facilitate their delivery to BamA β-barrel and the subsequent assembly/integration into OM. The C-terminal domain of BamD is crucial for its interaction with BamA, BamC, and BamE proteins (Voulhoux et al., 2003; Gatzeva-Topalova et al., 2010; Jansen et al., 2015; Bergal et al., 2016; Fleming et al., 2016). BamBCE individually are dispensable for cell viability, but their pair wise absence severely compromises cell growth and OMP biogenesis through the β-barrel of BamA (Sklar et al., 2007; Tellez and Misra, 2012).

Previous studies show that BamA and BamD can be reconstituted into a functional complex *in vitro*. It has been demonstrated that BamA and BamD function independently whereas in a coordinated manner (Hagan et al., 2010). POTRA domain 5 of BamA protein is required for interacting with BamD (Sinnige et al., 2015). Deletion analysis revealed that POTRA domains 3, 4, and 5 of BamA are essential for cell viability of *E. coli* (Kim et al., 2007). The interaction between BamA and BamD is also critical for BamA folding which is OMP as well. BamD can bind to the β-barrel domain of BamA but not POTRA domain *in vitro* when BamA is unfolded. Outcompeting the interaction between BamA and BamD for peptide derived from BamA’s β-barrel domain inhibits BamA assembly *in vitro* and is also toxic *in vivo* (Hagan et al., 2015). In BamD-deleted cells, the folding of BamA and OMPs decrease (Misra et al., 2015). Therefore, BamA and BamD interact with each other *in vitro* and *in vivo*, and this interaction is important for OMPs folding, OM localization, and bacteria survival.

In recent years, a collection of compounds that disrupts OM structure by targeting BamA or BamD have been identified, which present with promising anti-bacterial potential (Hagan et al., 2015; Hart et al., 2019b; Imai et al., 2019). These results give us implications that BAM complex is an effective and attractive target for developing novel antibiotics. In view of the important role of BamA–D interaction in OMPs folding, here, we established a yeast two-hybrid (Y2H) screening system to identify small molecules that could block the interaction between BamA and BamD in Gram-negative bacteria *E. coli.* Based on this screening, we identified a compound, IMB-H4, which disrupts the interaction between BamA and BamD and shows potent anti-bacterial activity with low toxicity to eukaryotic cells.

## Materials and Methods

### Yeast Two-Hybrid (Y2H) Assay

The Y2H system was purchased from Clontech (Arizona, United States) which includes AH109 strain, pGBKT (activation domain, AD), pGADT7 (DNA binding domain, BD), and control plasmids of pAD-T, pBD-53, and pBD-lam. The construction of Y2H system was performed as described (Wang et al., 2018). In briefly, the DNA fragments of *BamA* and *BamD* genes were amplified by PCR from the genome of *E. coli* (ATCC 25922 strain) and primers were listed in Supplementary Table S1. Four plasmids, pAD-BamA, pBD-BamD, pAD-BamD, and pBD-BamA were constructed and co-transferred into AH109 yeast strain to get AH109 (pAD-BamA + pBD-BamD) and AH109 (pAD-BamD + pBD-BamA). Strains AH109 (pAD + pBD-BamD) and AH109 (pAD-BamA + pBD) were constructed to detect self-activation. Strains AH109 (pAD-T + pBD-lam) and AH109 (pAD-T + pBD-53) were used as negative control and positive control, respectively. The positive transformants were selected by incubation on synthetic dropout (SD) plates (Clontech).

Positive transformants were confirmed by β-galactosidase (β-gal) activity analysis. The qualitative analysis of β-gal activity was performed as described (Lin et al., 2012). Quantification of β-gal activity is determined by β-gal assay kit (GENMED SCIENTIFICS INC., United States). Analysis was carried out according to equation: 1000 × A_420_/(t × V × OD_600_). In this equation, t is the incubation time (min) and V is the volume of cell cultures used for the assay (mL). The experiments were repeated three times. The expressions of BamA and BamD in AH109 cells were examined by western blotting using anti-Myc and anti-HA monoclonal antibodies (Beijing ComWin Biotech Co. Ltd., Beijing, China).

### Compound Library Screen

A library combining both synthetic (from Enamine) and natural products (from the Institute of Medicinal Biotechnology) which result with 25,000 compounds in total were screened. The screening assays were performed as described (Lin et al., 2012). Fresh AH109 (pAD-BamA + pBD-BamD) or AH109 (pAD-T + pBD-53) cells (OD600 = 0.8) were diluted 100-fold in SD/-Leu-Trp-Ade-His; 198 μL dilution and 2 μL compound were added to each well. The final concentration of each compound is 50 μg/mL in 0.1% DMSO. Yeast cells were cultured at 30°C for 2–3 days, and growth inhibition was analyzed afterward.

### Expression and Purification of Recombinant Proteins

For His-fusion and GST-fusion plasmids construction, the DNA fragment of *BamA* and *BamD* genes were amplified from the genome of *E. coli* (ATCC 25922 strain) and primers are listed in Supplementary Table S1. The PCR fragments were inserted into pET30a vector to generate recombinant proteins with a 6 × His-tag at C terminal. Meanwhile, the PCR fragments were inserted into pGEX-4T-1 expression vector to generate recombinant BamA with a GST-tag at N terminal. All constructs were sequenced for confirmation.

The expression of recombinant proteins and GST were induced by 0.5 mM IPTG in *E. coli* BL21 (DE3) (OD_600_ = 0.6) after overnight incubation at 20°C. Cells were collected by centrifugation at 5000 × *g* for 10 min at 4°C, and then suspended in lysate buffer and disrupted by Constant Systems (Constant Systems Ltd., United Kingdom). After further centrifugation at 12,000 × *g* at 4°C for 60 min, the supernatants were loaded onto a 1 mL column of His-Trap FF or GST-Trap 4B (GE Healthcare) pretreated with binding buffer. For His-tagged protein, unbound proteins were eluted with washing buffer, while bound proteins were eluted using elution buffer. But for purity of GST and GST-tagged BamA, washing buffer was not needed. Purified proteins were desalted using Amicon Ultra-15 Centrifugal Filter Units (Millipore, Maryland, United States). The purified proteins were verified by western blotting using anti-His or anti-GST antibody (Com Win Biotech Co., Beijing, China). Protein concentrations were determined by Bradford assay.

BamA proteins formed inclusion bodies when being overexpressed in BL21 cells. In order to obtain soluble proteins, all buffers used for the purification of His-tagged BamA proteins contain 8 M urea. Instead, for the preparation of GST-tagged BamA, 8 M urea affects the binding of GST-tagged proteins to the column, and only 2 M urea was added to the lysate buffer. The recipes for all buffers are listed in Supplementary Table S2.

### GST Pull-Down Assay

*In vitro* interruption of BamA-BamD interaction by IMB-H4 was analyzed by GST pull-down assay as described (Wang et al., 2018). In briefly, 4 μg/mL GST-tagged BamA was incubated with 30 μL glutathione sepharose beads in working buffer (GE Healthcare) for 2 h at 4°C. Unbound proteins in supernatant after centrifugation were removed while the beads were then suspended in working buffer containing 4 μg/mL His-tagged BamD, together with multiple concentrations of IMB-H4 (from 0 to 5 μg/mL with 1% DMSO). A certain proportion of the samples were separated from the reaction mixtures as input and the rest were incubated at 4°C for 4 h. Bound-proteins were detected by western blotting with anti-His and anti-GST antibodies (Beijing ComWin Biotech Co., Beijing, China). Protein bands were developed by HRP-conjugated secondary antibody and band intensity was quantified using ImageJ. GST-tagged BamA was replaced by GST-tag protein as the negative control.

### Biolayer Interferometry (BLI)

The binding of BamA and BamD to IMB-H4 was measured by biolayer interferometry (BLI) according to protocols described previously (Sultana and Lee, 2015). Briefly, His-tagged BamA or BamD was biotinylated using EZ link sulfo-NHS-LC-biotinylation kit (Thermo Pierce). All Super Streptavidin (SSA) biosensors were hydrated in BLI rehydration buffer for 10 min. Biotinylated BamA or BamD was diluted in BLI kinetics buffer to a final concentration of 20 μg/mL and immobilized onto an SSA-biosensor for 10 min. Compound IMB-H4 was prepared in BLI kinetics buffer with multiple concentrations and applied to BamA or BamD for 60 or 120 s. Subsequently, the SSA-biosensor was immersed into BLI kinetics buffer for 60 s to dissociate IMB-H4. Three negative controls were included: BLI kinetics buffer without IMB-H4 being associated to BamA or BamD, different concentrations of compound IMB-H4 in BLI kinetics buffer, or only BLI kinetics against SSA-biosensors biotinylated without BamA or BamD immobilization, to detect non-specific binding. The data were analyzed, sensor grams step corrected, reference corrected, and fit globally to a 1:1 binding model. The equilibrium dissociation constant (Kd) and *R*^2^ were calculated using the Octet Analysis software suite (ForteBio Data Analysis 9.0). All experiments were performed in triplicate.

### Scanning Electron Microscope (SEM) and Transmission Electron Microscopy (TEM)

*Escherichia coli* (ATCC 25922 strain) cell cultures in log-phase were diluted to 5 × 10^6^ CFU/mL in LB medium and treated with 5 μg/mL IMB-H4 for 12 h. Cells were treated and prepared for scanning electron microscope (SEM) and transmission electron microscopy (TEM) as described previously (Zhang et al., 2019). In briefly, cells were first fixed with 2.5% glutaraldehyde and then with 1% osmium tetroxide in sodium cacodylate buffer for 2 h. The fixed cells were dehydrated with serial increasing concentrations of ethanol. Some samples were infiltrated with araldite resin and visualized at 80 kV on a JEM-1400 TEM from Japan Electronics Co. Ltd. (JEOL). Some samples were precooled for 2 h at −20°C and then dried for 12 h by a freeze dryer (Han Mei Ecology Instrument Co., Ltd., Beijing, China). The dehydrated specimen was coated with gold-palladium and examined on FE-SEM (Regulus 8100, Hitachi, Japan).

### *In vitro* Accumulation of Ethidium Bromide (EtBr)

*Escherichia coli* (ATCC 25922 strain) cells were treated with IMB-H4 (from 0.03125 to 2.5 μg/mL) or DMSO (0.1%) for 12 h in LB medium with the density of 5 × 10^6^ CFU/mL. 100 μL culture were added into each well of black microtiter plates with clear bottoms, and then ethidium bromide (EtBr) was added to a final concentration of 4 μg/mL. The relative fluorescence intensity was immediately recorded every 60 s for 10 min using fluorescence plate reader (Perkin Elmer EnSpire^®^ 2300, United States). The emission and excitation wavelength were 530 and 600 nm, respectively.

### Out Membrane Fractions Isolation

Cells of *E. coli* ATCC 25922 strain were treated with DMSO or IMB-H4 (1.25–5 μg/mL) for 12 h and then collected by centrifugation. The inner membrane and OM were separated by discontinuous sucrose density gradient centrifugation as described previously (Wu et al., 2005). In brief, cells were treated with Lysozyme (2 mg/mL) to convert to spheroplasts. The spheroplasts were then disrupted by sonication and the final lysate was added to the top of a preliminary sucrose gradient containing 1.0 mL 25% (wt/wt) sucrose layered over 0.3 mL 65% (wt/wt) sucrose. Samples were centrifuged for 4 h at 55,000 r/min, 4°C (Beckman Optima L7 ultracentrifuge). The bottom 1 mL fraction was collected and mixed with 1.4 mL EDTA (5 mM). Step gradients were prepared with the following concentrations of sucrose from bottom to top: 0.2 mL 65% sucrose, 0.2 mL 55% sucrose, 0.4 mL 50% sucrose, 0.8 mL 45% sucrose, 0.8 mL 40% sucrose, 0.8 mL 35% sucrose, and 0.5 mL 30% sucrose. Gradients were centrifuged for 17 h at 36,000 r/min. From top to bottom, every 200 μL was taken and all samples were analyzed by western blot with anti-OmpC and anti-OmpA antibodies (Biorbyt, San Francisco, CA, United States).

### SDS-PAGE

SDS-PAGE has been used to assess OMP folding, as it can distinguish between folded and unfolded protein populations (Inouye and Yee, 1973; Nakamura and Mizushima, 1976; Hagan et al., 2013). In this study, SDS-PAGE was used to analyze the folding of BamA proteins, which are either purified from bacteria or expressed in yeast cells for Y2H assay. The purified His-tagged and GST-tagged BamA protein were diluted with TBS (pH 8). We took aliquots and 2x SDS sample loading buffer was added, the aliquots were boiled at 100°C for 5 min, or unboiled. To enable protein folding, the diluted protein samples were treated with 0.5% *N,N*-dimethyldodecylamine *N*-oxide (LADO) at 25°C for 1 h. The folding was stopped with 2x SDS sample loading buffer. Half of each sample was boiled. All the samples with different treatment were resolved on 10% SDS-PAGE at 120V for 120 min at 4°C. After electrophoresis, the gels were stained with Coomassie Blue and scanned used a gel-doc system (FluroChem M, ProteinSimple, United States).

To detect HA-tagged BamA in yeast cells, the cultures of strain AH109 (pAD-BamA + pBD-BamD) in the mid-log phase (OD_600_ = 0.5) were harvested and then resuspended in distilled water and then lyzed by freeze/thaw cycles. After centrifugation (12,000 r/min, 10 min, 4°C), 2x SDS sample loading buffer was added to the supernatant. Only half of each sample was heated. All the heated and unheated samples were resolved on SDS-PAGE and followed by western-blotting analysis using anti-HA monoclonal antibodies.

### The Inhibition of *E. coli* Strain and Other Gram-Negative Bacteria Strains Growth

Growth inhibition of Gram-negative bacteria strains by IMB-H4 was determined according to guidelines of the Clinical and Laboratory Standards Institute (CLSI). Cells in mid-log phase were diluted with Mueller–Hinton broth (5 × 10^5^ CFU/mL) with multiple concentrations of IMB-H4 (ranging from 1 to 64 μg/mL). The minimum inhibitory concentration (MIC) was defined as the lowest drug concentration that inhibits cell growth.

### The Mode of Action

Cell cultures of *E. coli* strain (ATCC 25922) in log-phase were diluted to 1 × 10^6^ CFU/mL and cultured in LB medium containing IMB-H4 (from 0 to 8 × MIC). Bacteria were collected every hour and spread on to LB plates after serial dilution. The plates were placed in 37°C incubator for 24 h and the number of colonies was counted.

### Synergistic Effect of IMB-H4 on Other Antibiotics

The synergistic effect of compounds *in vitro* was determined using checkerboard assay. Growth inhibition of *E. coli* strain (ATCC 25922) by compounds was determined according to CLSI. The MIC was defined as the lowest drug concentration that inhibits cell growth. The fractional inhibitory concentration index (FICI) is calculated according to the following formula: FICI = (MIC_drug A in combination_)/(MIC_drug A alone_) + (MIC_drug B in combination_)/(MIC_drug B alone_). FICI ≤ 0.5 was considered as synergistic effect.

### Cytotoxicity Assay

Hela cells were diluted in DMEM medium with 10% FBS and added into 96 well plates with 5 × 10^3^ cells/well in triplicates. Cells in log-phase were then incubated in DEME medium without FBS containing gradient concentrations of IMB-H4 (ranging from 3.125 to 100 μg/mL). After incubation for 48 h, the medium was aspirated, and fresh medium was added. After incubation for 24 h at 37°C, MTT reagent was added and further incubated for 4 h. The absorbance was measured at 570 nm after addition of 50 μL of DMSO. The IC_50_ values were calculated based on a concentration–response curve.

### Statistical Analysis

All data analyses were performed using GraphPad Prism, version 5, software for Windows (GraphPad Software, San Diego, CA, United States). The *t*-test was used to determine the difference between treatment groups and the control. A *P*-value < 0.05 (two-tailed) is considered statistically significant. The data were presented as mean ± SD values.

## Results

### Confirmation of Interaction Between *E. coli* BamA and BamD Proteins Using Yeast Two-Hybrid Assay

**FIGURE 1:**
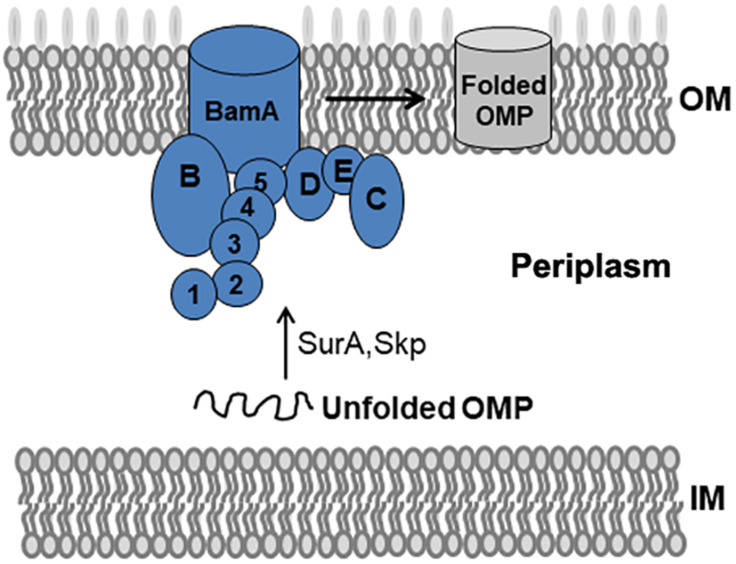
BAM complex model and its mediated folding of OMPs in *E. coli*. The BAM complex consists of BamA, an OM β-barrel protein with five N-terminal POTRA domains, and the lipoproteins BamB–E. Nascent OMPs are translocated across the inner membrane (IM) into the periplasm. Chaperones such as SurA and Skp recognize unfolded OMPs in the periplasm and transport them to the BAM complex. The BAM complex receives, folds, and inserts OMPs at the OM.

**FIGURE 2:**
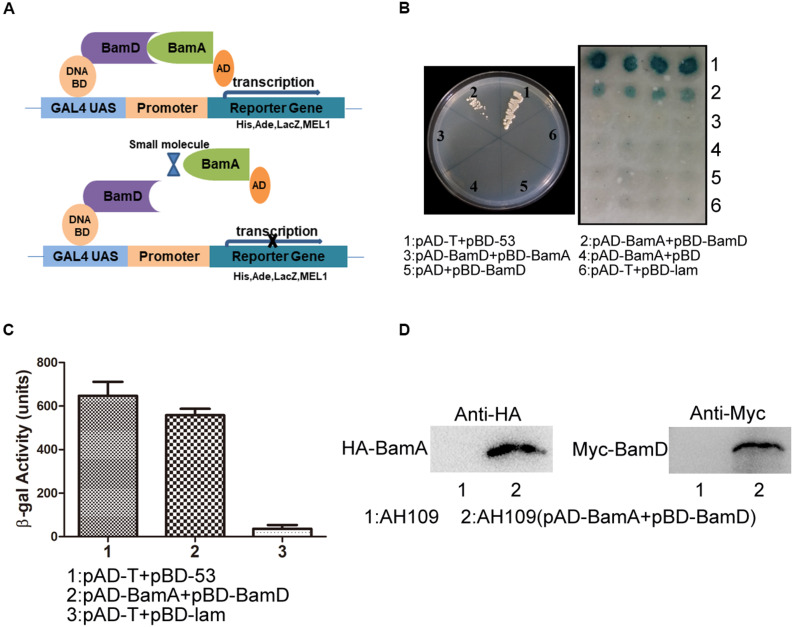
Construction of the Y2H system to detect BamA–BamD interaction. **(A)** The strategy for high-throughput screening using the Y2H system. BamA–BamD interaction induces the expression of reporter genes, ADE2, HIS3, and LacZ. The compounds that disrupt BamA–BamD interaction prevent the expression of these reporter genes. **(B)** The growth and LacZ-dependent color change of yeast cells with various combinations of BD and AD fusions. The left panel shows the growth of yeast cells with indicated plasmids on an SD/-Leu-Trp-Ade-His dropout plate. The right panel shows the β-gal activity of indicated strains in SD/-Leu-Trp dropout plate. **(C)** Quantification of β-gal activity in yeast cells. The results are the average from triplicated assays. **(D)** The expression of BamA and BamD proteins in yeast cells. The expression was detected using anti-HA and anti-Myc antibodies.

The BAM complex recruits nascent OMP-chaperone components and efficiently catalyzes OMP insertion and assembly in OM (Figure 1). In the BAM complex, the interaction between BamA and BamD is crucial for rapid integration of OMPs to OM. In this study, we established a Y2H system to confirm BamA–BamD interaction and further identify potential compounds that could specifically block this interaction (Figure 2A). In AH109 cells, the transcription of three reporter genes *ADE2*, *HIS3*, and *LacZ* can be activated by the interaction between BamA and BamD, which can be validated by detecting β-gal activity after growing yeast cells on SD/-Leu-Trp-Ade-His plate.

AH109 (pAD-BamA + pBD-BamD) and the positive control strain AH109 (pAD-T + pBD-53) grew well on SD/-Leu-Trp-Ade-His plate and both exhibited with positive β-gal activity, indicating the existence of interaction between BamA and BamD. False-positive and self-activation were excluded because the negative control strain AH109 (pAD-T + pBD-lam) and AH109 cells expressing either BamA or BamD alone, neither grew on SD/-Leu-Trp-Ade-His plate nor showed β-gal activity. Surprisingly, AH109 (pAD-BamD + pBD-BamA) cells showed negative result for the Y2H assay (Figures 2B,C). We reason that the fusion of these proteins may prevent their interaction.

The expression of BamA and BamD proteins in yeast cells was examined by western blot (Figure 2D). Collectively, the Y2H system was constructed successfully to determine interaction between BamA and BamD, which could be used as a readout for interaction inhibitor screening.

### BamA–BamD Interaction-Disrupting Compound Screening Using Y2H

**FIGURE 3:**
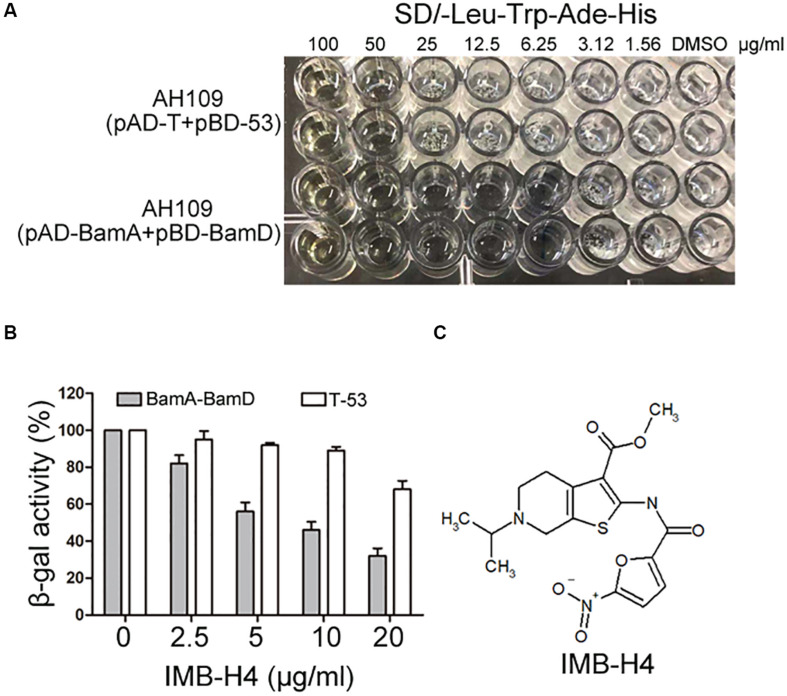
Identification of potential compound that disrupts BamA–BamD interaction. **(A)** Growth inhibition of yeast cells by IMB-H4. Yeast strains with indicated plasmids seeded in 96-well plates were incubated in SD/-Leu-Trp-Ade-His dropout medium with duplicates. The final concentration of IMB-H4 was from 5 to 100 μg/mL. **(B)** The inhibition of β-gal activity of AH109 (pAD-BamA + pBD-BamD) cells by IMB-H4 at multiple concentrations. Strain AH109 (pAD-T + pBD-53) was applied as a control. Values represent the ratio of β-gal activity of cells treated with compounds over that of untreated cells. The results are the average units from triplicated assays. **(C)** Structure of compound IMB-H4.

The growth of AH109 (pAD-BamA + pBD-BamD) and AH109 (pAD-T + pBD-53) seeded in 96-well plates in SD/-Leu-Trp-Ade-His dropout medium were detected in the presence of compounds at 50 μg/mL. AH109 (pAD-T + pBD-53) was used as a control to exclude possible compounds that could block Gal4 expression or showed anti-fungal activity to inhibit the growth of AH109 (pAD-BamA + pBD-BamD). Compounds that could specifically inhibit the growth of AH109 (pAD-BamA + pBD-BamD), but not AH109 (pAD-T + pBD-53) were selected. For those compounds that exhibited with comparable growth inhibition activities for both two strains at 50 μg/mL, their MICs were further determined. Compounds with MIC for AH109 (pAD-BamA + pBD-BamD) less than half of that of AH109 (pAD-T + pBD-53) were selected. After initial screening, five compounds were selected from 25,000 compounds. A quantitative β-gal assay with multiple concentrations of the selected compounds was performed to further confirm if the identified compounds could selectively block BamA–BamD interaction. Among the five candidates, the MIC of IMB-H4 for AH109 (pAD-BamA + pBD-BamD) was 6.25 μg/mL but 50 μg/mL for AH109 (pAD-T + pBD-53) (Figure 3A). Furthermore, IMB-H4 inhibited the β-gal activity of AH109 (pAD-BamA + pBD-BamD) in a dose-dependent manner. The β-gal activity of strain AH109 (pAD-T + pBD-53) was also inhibited by this compound, whereas the inhibition is less efficient (Figure 3B). Therefore, IMB-H4 was selected for further investigation and its structure is shown in Figure 3C.

### IMB-H4 Disrupts BamA–BamD Interaction by Binding to BamA

**FIGURE 4:**
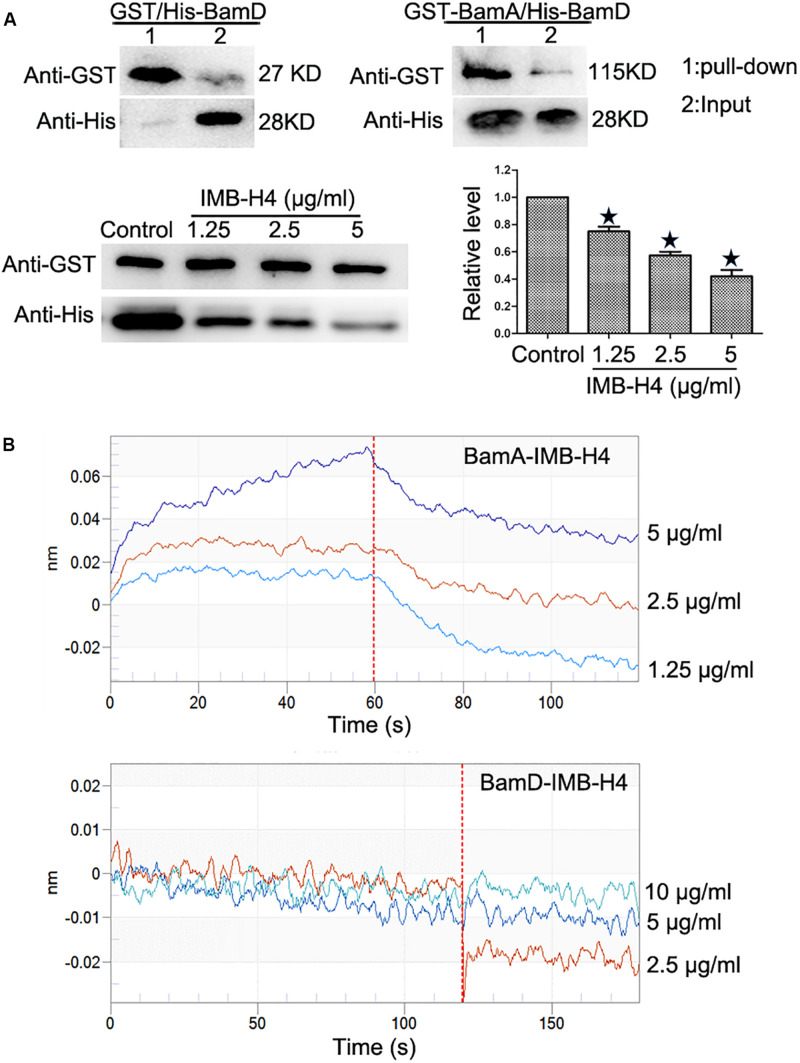
Disruption of BamA–BamD protein interaction by IMB-H4. **(A)** Compound IMB-H4 blocks BamA–BamD interaction. For the detection of BamA–BamD interaction, the purified GST-tagged BamA or GST were bound to glutathione-Sepharose beads, and then incubated with purified His-tagged BamD. In parallel, IMB-H4 was incubated with GST-tagged BamA proteins bound to glutathione-Sepharose beads, and then His-tagged BamD was added to detect if IMB-H4 disrupts BamA–BamD interaction. Equal volume of DMSO was added as a control. Western blotting bands of resulting pull-down products are shown and the levels are normalized to GST-BamA. Data are shown as mean ± SD, *n* = 3. *p < 0.05 vs DMSO group. **(B)** IMB-H4 binds to BamA. The binding of BamA and BamD to IMB-H4 was measured by BLI.

Recombinant proteins were expressed in *E. coli* BL21 and purified (Supplementary Figure S1). GST pull-down assay was implemented to detect the disruption of BamA–BamD interaction by IMB-H4 *in vitro*. In this assay, GST-BamA fusion protein or GST protein was used as a bait to incubate with His-BamD and the resulting pull-down products were determined by western blotting. A His-BamD protein band (anti-His) was found in the GST-BamA pull-down products but not in the products pull-down with GST protein, which confirmed the *in vitro* interaction between BamA and BamD. GST-BamA protein and His-BamD were incubated with IMB-H4 and then the level of His-BamD protein in the pull-down products was analyzed. DMSO treated samples was used as a negative control. Strikingly, IMB-H4 significantly decreased the level of His-BamD in a dose-dependent manner (Figure 4A).

Next, the binding of compound IMB-H4 to BamA and BamD proteins was assessed by BLI assay. The results demonstrated that IMB-H4 binds to BamA in a dose-dependent manner (*R*^2^ = 0.999211093, Kd = 3.90E^–6^), but IMB-H4 did not show any binding to BamD (Figure 4B). Collectively, these data indicate that compound IMB-H4 blocks BamA–BamD interaction *in vitro* by binding to BamA.

### The Effects of IMB-H4 on the OMPs and OM Structure

**FIGURE 5:**
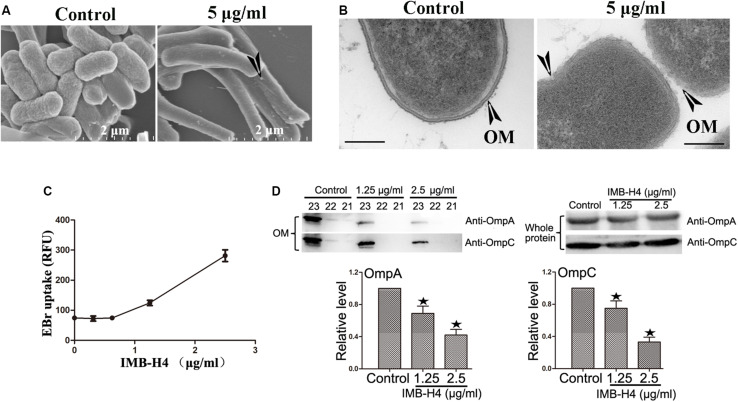
*E. coli* treated with IMB-H4 shows out membrane damage and OMPs reduction in OM. *E. coli* cells were treated with compound IMB-H4 or DMSO (1%) for 12 h and then collected for morphological assessment. **(A)** Observation of morphological alterations of *E. coli* treated with IMB-H4 for 12 h by scanning electron microscope (SEM). **(B)** Observation of morphological alteration of *E. coli* treated with IMB-H4 for 12 h by transmission electron microscopy (TEM). **(C)** *E. coli* were treated with IMB-H4 (ranging from 0.3725 to 2.5 μg/mL) or DMSO (0.1%) for 12 h. EtBr was then added into the medium to a final concentration of 4 μg/mL. The relative fluorescence intensity was shown. **(D)** *E. coli* were treated with IMB-H4 (1.25 or 2.5 μg/mL) or DMSO (0.1%) for 12 h. Distribution of OmpA and OmpC in the OM and whole protein. *E. coli* were treated as described above and then the whole protein or the outer membrane fraction was isolated separately. The levels of OmpA and OmpC were detected using western blotting with anti-OmpA or anti-OmpC antibody. Western blotting bands of the outer membrane fraction are shown and the levels are normalized to whole protein. Data are shown as mean ± SD, *n* = 3. *p < 0.05 vs DMSO group.

The activity of BAM complex is essential to maintain the barrier function of OM and impairment of this function could disrupt the integrity of OM (Urfer et al., 2016; McCabe et al., 2017; Hart et al., 2019a, b; Imai et al., 2019). When IMB-H4-treated *E. coli* cells were examined by SEM, the most notable feature was the appearance of knob-like structures over cell surface, which was not observed in control cells (Figure 5A). The perturbation in membrane morphology was revealed by TEM. Comparing to DMSO-treated cells, *E. coli* cells treated with 5 μg/mL IMB-H4 showed distinct ruptures in the OM (Figure 5B). To further determine the effect of IMB-H4 on OM integrity, we also measured EtBr permeability in *E. coli*, which cannot penetrate an intact OM. IMB-H4 treatment caused a dose-dependent increase in EtBr uptake (Figure 5C). These results indicate a dramatic effect of IMB-H4 on the integrity of OM.

β-barrel assembly machinery complex plays a prominent role in the folding process of OMPs, such as OmpA and OmpC. The absence of BamA and BamD proteins results in a decreased distribution of OMPs in the OM (Malinverni et al., 2006; Imai et al., 2019). Therefore, the effect of IMB-H4 on OmpA and OmpC distribution in *E. coli* ATCC25922 cells was investigated. OM fractions from IMB-H4-treated bacteria and control cells were isolated by differential centrifugation, and further analyzed by western blotting. We took a series of samples after density gradient centrifugation, and then analyzed all of them. The results showed that OmpA and OmpC were concentrated in fractions from sample 21 to 23 in the both of IMB-H4 treated and the control groups (Supplementary Figure S2). The total protein levels of OmpA and OmpC remained unchanged, but these proteins decreased significantly in the OM fraction after IMB-H4 treatment compared with untreated cells (Figure 5D).

### IMB-H4 Disrupts BamA–BamD Interaction *in vivo*

Because BamA and BamD are essential for the growth of *E. coli* and their interaction is important for their function, we speculated that disruption of this interaction would inhibit the growth of *E. coli* cells. IMB-H4 showed an MIC of 4 μg/mL for *E. coli* ATCC 25922 strain. The MICs of IMB-H4 for the clinical isolated *E. coli* strains ranged from 4 to 32 μg/mL (Table 1).

**TABLE 1:** The MICs (μg/mL) of IMB-H4 and other antibiotics against *E. coli* strains.

**Antibiotics**	***E. coli* 1**	***E. coli* 2**	***E. coli* 3**	***E. coli* 4**	***E. coli* 5**	***E. coli* 6**	***E. coli* 7**	***E. coli* 8**	***E. coli* 10**	**ATCC 25922**
IMB-H4	16	32	8	4	32	16	16	8	32	4
Cefipime	<0.5	256	1024	512	<0.5	>1024	1024	1024	256	<0.5
Cefoxitin	2	512	2	1024	1	512	512	512	512	2
Meropenem	<0.5	64	<0.5	<0.5	<0.5	32	64	128	32	<0.5
Gentamicin	16	64	64	128	1	64	>1024	32	>1024	<0.5
Minocycline	2	8	4	2	2	2	4	8	16	<0.5
Levofloxacin	1	64	<0.5	16	<0.5	16	16	32	16	<0.5
Colistin	256	4	2	2	4	4	1	4	8	<0.5
Ticarcillin	128	>1024	>1024	>1024	1024	>1024	1024	>1024	>1024	4

**E. coli* 1–10 are all clinical drug-resistant strains.*

BamA and BamD proteins are evolutionarily conserved in Gram-negative bacteria (Voulhoux and Tommassen, 2004; Wu et al., 2005; Malinverni et al., 2006; Volokhina et al., 2009; Anwari et al., 2012; Webb et al., 2012; Yu and Lu, 2019). Agents targeting BamA–BamD interaction should show antibacterial activity against other Gram-negative bacteria (Urfer et al., 2016; Storek et al., 2018; Choi and Lee, 2019). We found that IMB-H4 showed growth inhibition to *Klebsiella pneumoniae*, *Pseudomonas aeruginosa*, and *Acinetobacter baumannii* (Table 2). The MICs of IMB-H4 against *P. aeruginosa* and *A. baumannii* were 4 μg/mL, but the MIC was 32 μg/mL for *K. pneumonia*. We also examined the growth inhibition of IMB-H4 to human cells. The IC_50_ for Hela cells was 76.5 μg/mL, indicating that human cells are less sensitive to IMB-H4 compared with *E. coli*.

**TABLE 2:** The MICs (μg/mL) of IMB-H4 against other Gram-negative strains.

	***K. pneumoniae* ATCC 700603**	***P. aeruginosa* ATCC 19606**	***A. baumannii* PA01**
IMB-H4	32	4	4
Levofloxacin	0.5	0.125	2

**FIGURE 6:**
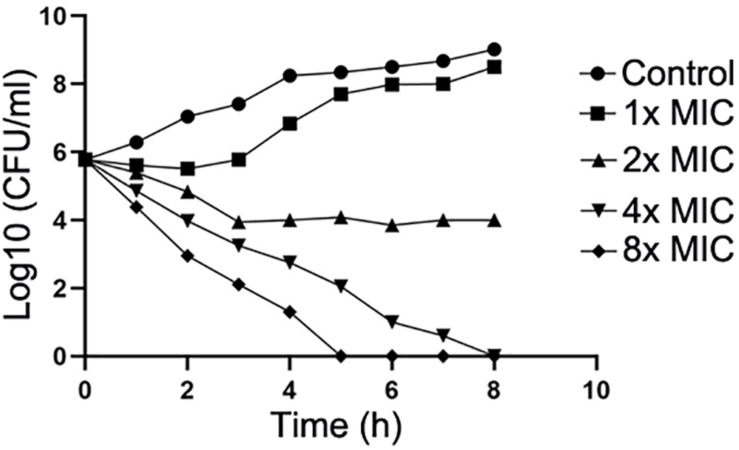
Compound IMB-H4 exhibits bacteriostatic and bactericidal activities. The MIC of IMB-H4 for *E. coli* ATCC 25922 is 4 μg/mL. The colony number was counted after incubation in the presence of IMB-H4 at one-, two-, four-, and eight-fold of MICs.

Next, the action mode of compound IMB-H4 against *E. coli* was assessed by examining the growth of *E. coli* treated with multiple concentrations of this compound. IMB-H4 showed bacteriostatic effect and the number of colonies increased very slowly in the presence of 1 × MIC but showed bactericidal activity at 2 × MIC and the activity was increased significantly when the concentration reached to 4 × MIC or higher (Figure 6).

If IMB-H4 inhibits the growth of *E. coli* by binding to BamA, then high level of BamA protein expression should reduce the antibacterial activity of IMB-H4. To test this hypothesis, BamA and BamD were overexpressed in *E. coli* BL21. The MICs of IMB-H4 for *E. coli* BL21 with control vector or BamD overexpressing vector were all 4 μg/mL; however, the MIC for *E. coli* BL21 with BamA overexpressing vector was 16 μg/mL. To clarify if the increase of MIC is specific to IMB-H4, the MICs of Ciprofloxacin and Ceftriaxone Sodium against the *E. coli* BL21 overexpressing BamA or BamD were detected. The bacteria strains overexpressing these two proteins exhibited comparable sensitivities to Ciprofloxacin and Ceftriaxone Sodium, and their MICs were 1.25 and 0.0125 μg/mL, respectively. The experiment was repeated for six times and produced consistent results.

The OM barrier confers bacteria on highly selective permeability, thereby precluding the clinic use of many antibiotics. *E. coli* cells showed OM rupture when treated with IMB-H4, suggesting that IMB-H4 may enhance the antibacterial activity of other antibiotics by increasing OM permeability. Indeed, IMB-H4 showed synergistic antibacterial activity with Gentamicin, Polymyxin B, and Vancomycin against *E. coli* ATCC25922 (Table 3). In particular, Vancomycin did not inhibit the growth of *E. coli* until 200 μg/mL, but its MIC reduced to 50 μg/mL in the presence of IMB-H4. Collectively, BamA is likely to be the target of IMB-H4 and the disruption of BamA–BamD interaction may contribute to its bacteriostatic activity.

**TABLE 3:** The synergetic effect of IMB-H4 with other antibiotics against *E. coli*.

**Compounds**	**MIC (μg/mL)**		**MIC of IMB-H4 (μg/mL)**	**FICI**
	**Alone**	**Combination**	**Combination**	
Polymycin B	0.25	0.0625	1	0.5
Vancomycin	>100	50	1	<0.5
Gentamicin	12.5	1.5625	1	0.375
IMB-H4	4	–	–	–

*MIC is the minimum inhibitory concentration. FICI is fractional inhibitory concentration. FICI ≤ 0.5 was considered as synergistic effect.*

### Analyze the Activity of Other 5-Nitrofuran Derivatives Using the Yeast Two-Hybrid System

IMB-H4 is a derivative of 5-nitrofuran. To test whether such kind of compounds could also block the BamA and BamD interaction as IMB-H4 does, we assessed the MICs of three 5-nitrofuran derivatives furazolidone (FZ), nitrofurazone (NFZ), and nitrofurantoin (NIT) on the Y2H model. Among them, the MICs of FZ and NFZ for AH109 (pAD-BamA + pBD-BamD) were 50 μg/mL but 100 μg/mL for AH109 (pAD-T + pBD-53) in SD/-Leu-Trp-Ade-His dropout medium (Table 4). As for NIT, the same MIC was detected in both AH109 (pAD-BamA + pBD-BamD) and AH109 (pAD-T + pBD-53). Compared with IMB-H4, these three compounds did not show distinctive difference in the MICs against the two yeast strains.

**TABLE 4:** The MICs of antibiotics against the yeast two-hybrid models.

**Strains**	**MIC (μg/mL)**			
	**FZ**	**NFZ**	**NIT**	**IMB-H4**
AH109 (pAD-BamA + pBD-BamD)	50	100	50	6.25
AH109 (pAD-T + pBD-53)	100	100	100	50
ATCC25922	1	8	4	4

*FZ, furazolidone; NFZ, nitrofurazone; NIT, nitrofurantoin.*

## Discussion

The BAM complex localizes in the OM of Gram-negative bacteria. Two subunits in this complex, BamA and BamD, are essential for bacteria growth. In recent years, a wild variety of compounds targeting BamA or BamD have been identified to impair the OM and show promising antibacterial activity (Hagan et al., 2015; Hart et al., 2019b; Imai et al., 2019). In particular, darobactin, a newly reported large molecule shows anti-bacteria activity both *in vitro* and *in vivo* by targeting the lateral gate conformation of BamA, which is outside the OM (Imai et al., 2019). All these studies demonstrate the potential of BAM complex as a novel target for antibiotics to treat infections caused Gram-negative bacteria. However, no comprehensive high-throughput screening of antibacterial drugs targeting the BAM complex has been reported. In this study, we aimed at identifying compounds that target BamA–BamD interaction in *E. coli* by high-throughput screening using Y2H system. With this system, compound IMB-H4 was outlined as a potent candidate, and we further demonstrated that this compound blocks BamA–BamD interaction by a couple of assays, including GST pull-down, BLI, SEM, and TEM assay. More importantly, IMB-H4 showed potent inhibitory activity against *E. coli* ATCC 25922 strain, as well as some clinically isolated drug-resistant strains. In summary, we have identified IMB-H4 as an anti-*E. coli* compound that likely targets BamA–BamD interaction to inhibit bacterial growth.

The BamA protein can be divided into two regions: one is the soluble POTRA domains at N-terminus while the other region is membrane β-barrel domain at the C-terminus (Nikaido, 2003; Gatzeva-Topalova et al., 2010; Jansen et al., 2015; Bergal et al., 2016; Fleming et al., 2016). Previous studies show that the POTRA domain 5 is responsible for the recruitment of BamC, B, and D when using membrane pellet of whole cell lysates of *E. coli*, where the BAM complex assembles and functions (Wu et al., 2005; Kim et al., 2007). However, using an *in vitro* system, BamD directly binds to the C-terminal of β-barrel domain of unfolded BamA, but not the POTRA domain, and this interaction facilitates BamA folding (Hagan et al., 2015). In yeast cells where the two-hybrid system works, the efficiency of self-folding of the β-barrel region in BamA protein is relatively slow, and this region should be mainly in unfolded form. We detected the expression of BamA protein in yeast cells using SDS-PAGE, in which the folded and unfolded proteins migrate to different position. We found that BamA protein existed in an unfolded state (Supplementary Figure S3A). In addition, in GST pull-down and BLI assay, soluble BamA and BamD proteins were used to detect their interaction, and in this system, BamA likely present as unfolded form as high concentration of urea was used to prepare protein samples (Supplementary Figure S3B). The interaction between C-terminal of BamA and BamD is important for the folding of BamA, which facilitates the assembly of BAM complex. In addition, expression of a short peptide in the C-terminal of BamA β-barrel domain blocks BamA–D interaction *in vitro* and inhibits the growth of *E. coli* (Hagan et al., 2015). Therefore, we reason that IMB-H4 likely disrupts BamA–BamD interaction by binding to the C-terminal of the β-barrel domain of BamA which may impair BamA folding and contribute to the antibacterial activity of IMB-H4.

As a member of the superfamily of Omp85 proteins, BamA is highly conserved in Gram-negative bacterial species (Voulhoux et al., 2003; Heinz and Lithgow, 2014). For unfolded BamA, the conserved C-terminal β-signal within the β-barrel domain is responsible for its direct interaction with BamD (Kutik et al., 2008; Hagan et al., 2015). In this study, we established the Y2H system to detect BamA–BamD interaction and identified IMB-H4 as a potent inhibitor for this interaction. In addition, IMB-H4 showed growth inhibition to a variety of Gram-negative bacteria. We speculate that disruption of the interaction between BamA and BamD by IMB-H4 contributes to the growth inhibition of different Gram-negative bacteria. However, further analysis is needed to verify this possibility, including identification of the binding sites of IMB-H4 at BamA protein and the assessment of this binding site conservation among different bacteria. We also need to analyze whether the antibacterial activity of IMB-H4 against the other Gram-negative bacteria is due to the block of BamA–BamD interaction. On the other hand, although the interaction between BamA and BamD has been well established, the impact of protein folding on this interaction remains unclear. Moreover, the sequence of BamD is diverse across Gram-negative bacteria (Sandoval et al., 2011), and the influence of this difference on BamA–BamD interaction remains to be determined. In addition, Gram-negative bacteria may show different tolerance to the disruption of OM structure, thus it is not clear whether inhibitors of BamA–BamD interaction show similar antibacterial activity against different Gram-negative bacteria.

Mutation sites analysis of drug resistant strains is needed to define the *in vivo* target of a compound. We used high concentration of IMB-H4 to induce drug-resistant bacteria strains, but we only obtained a few colonies that showed 2 × MIC, and no mutation was detected in *BamA* and *BamD* gene in these mutants. The resistance may not be specific to IMB-H4, because these strains are also resistant to other antibiotics.

IMB-H4 is a derivative of 5-nitrofuran. Some 5-nitrofuran derivatives, such as FZ, NIT, and NFZ, have been used clinically for bacterial infections treatment since their introduction in 1940s and 1950s (Vass et al., 2008; Zhuge et al., 2018). In this research, we also detected the MICs of other 5-nitrofunran derivatives (FZ, NIT, and NFZ) against AH109 (pAD-BamA + pBD-BamD) and AH109 (pAD-T + pBD-53). Unlike IMB-H4, these three compounds did not show significant difference in the MICs against the two yeast strains, indicating that their target is unlikely BamA–BamD interaction. The MICs remain the same for bacterial strains. These results suggests that the antibacterial mechanism of IMB-H4 is likely different from other nitrofuran antibiotics used in clinic, and BamA is likely one of *in vivo* targets of IMB-H4.

## Conclusion

We have successfully established the Y2H system to screen inhibitors for BamA–BamD interaction and identified the compound IMB-H4 with antibacterial activity. However, more efforts are needed to validate the disruption of this interaction by IMB-H4 in cells, reveal the biding sites of IMB-H4 with BamA, and evaluate its antibacterial activity of *in vivo*.

## Data Availability Statement

All datasets generated for this study are included in the article/Supplementary Material.

## Author Contributions

YaL, XZ, NZ, and SS designed the research. YaL and XZ performed research. JZ determined the mode of action of IMB-H4 against *E. coli*. YuL contributed to the construction of Y2H system. XY tested the MIC of IMB-H4 against *E. coli*. MC contributed to the structural analysis. YW contributed by polishing the language. YaL, NZ, and SS drafted the manuscript.

## Conflict of Interest

The authors declare that the research was conducted in the absence of any commercial or financial relationships that could be construed as a potential conflict of interest.

## Supplementary Material

FIGURE S1 Expression of BamA and BamD proteins in *E. coli* cells. *E. coli* cells with fusion BamA or BamD plasmids were grown in the presence of induction agent IPTG, and the expression was detected by SDS-PAGE and stained by Coomassie Blue. **(A)** The expression and purification of His-BamA. **(B)** The expression of GST-BamA. **(C)** The purification of GST-BamA. **(D)** The expression and purification of His-BamD. **(E)** The expression and purification of GST tag.

FIGURE S2 Western blotting bands of the outer membrane fraction.

FIGURE S3 Analyze the folding of purified BamA and that expressed in Y2H system. **(A)** BamA expressed in unfolded form in Y2H system. AH109 (pAD-BamA + pBD-BamD) cultures were harvest and disrupt. The supernatant was heated or unheated in SDS sample buffer. The HA-BamA protein was determined using western blotting with anti-HA monoclonal antibodies. **(B)** The purified BamA protein was unfolded. SDS sample loading buffer was added to the purified BamA protein, and then heated or unheated. Proteins were separated by 10% SDS-PAGE and stained by Coomassie Blue.

TABLE S1 The primer pairs.

TABLE S2 The buffer used for protein purification.
