# Supplementary figures and images for: Identification of a Compound That Inhibits the Growth of Gram-Negative Bacteria by Blocking BamA–BamD Interaction
Source: Front Microbiol. 2020 Jun 19;11:1252. doi: 10.3389/fmicb.2020.01252 (PMC7316895; doi:10.3389/fmicb.2020.01252)

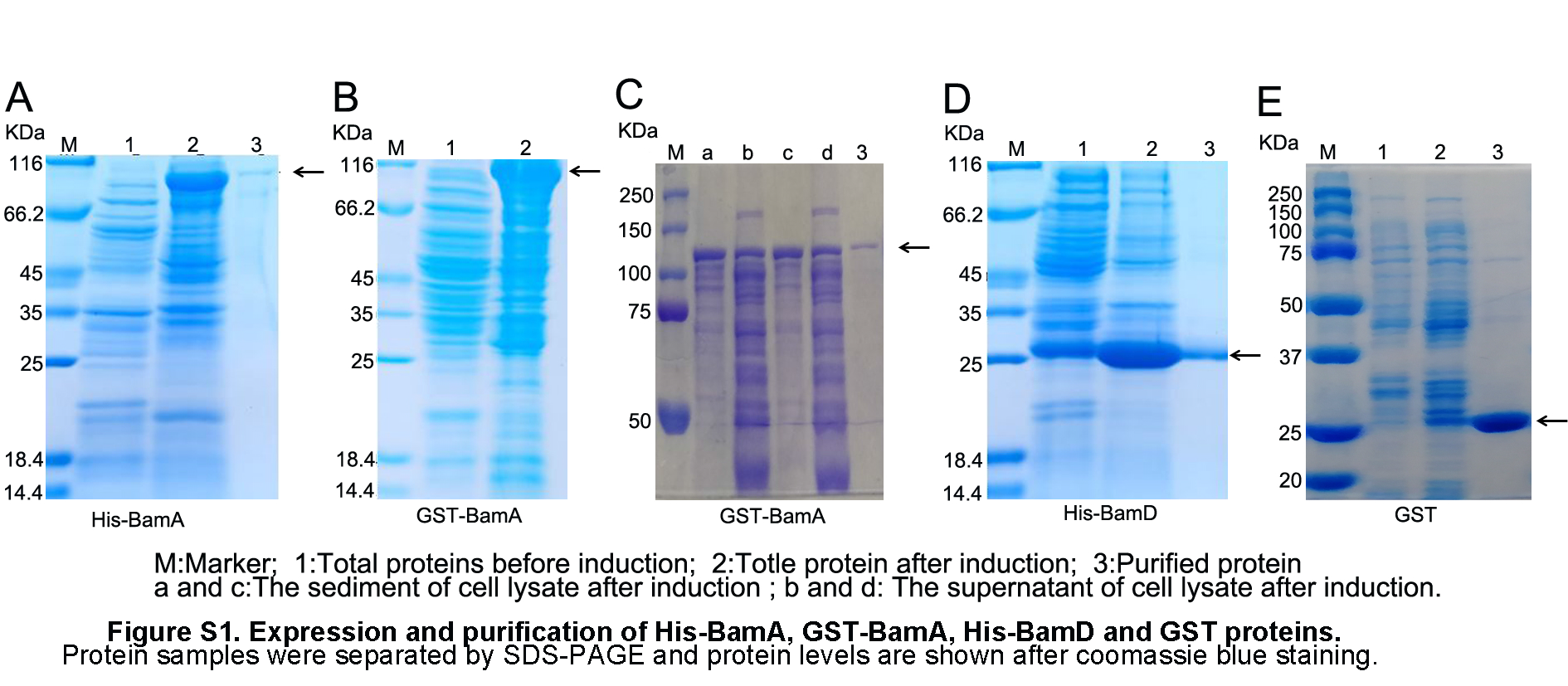

Supplement: FIGURE S1 — Expression of BamA and BamD proteins in E. coli cells. E. coli cells with fusion BamA or BamD plasmids were grown in the presence of induction agent IPTG, and the expression was detected by SDS-PAGE and stained by Coomassie Blue. (A) The expression and purification of His-BamA. (B) The expression of GST-BamA. (C) The purification of GST-BamA. (D) The expression and purification of His-BamD. (E) The expression and purification of GST tag. [file Image_1.TIFF]

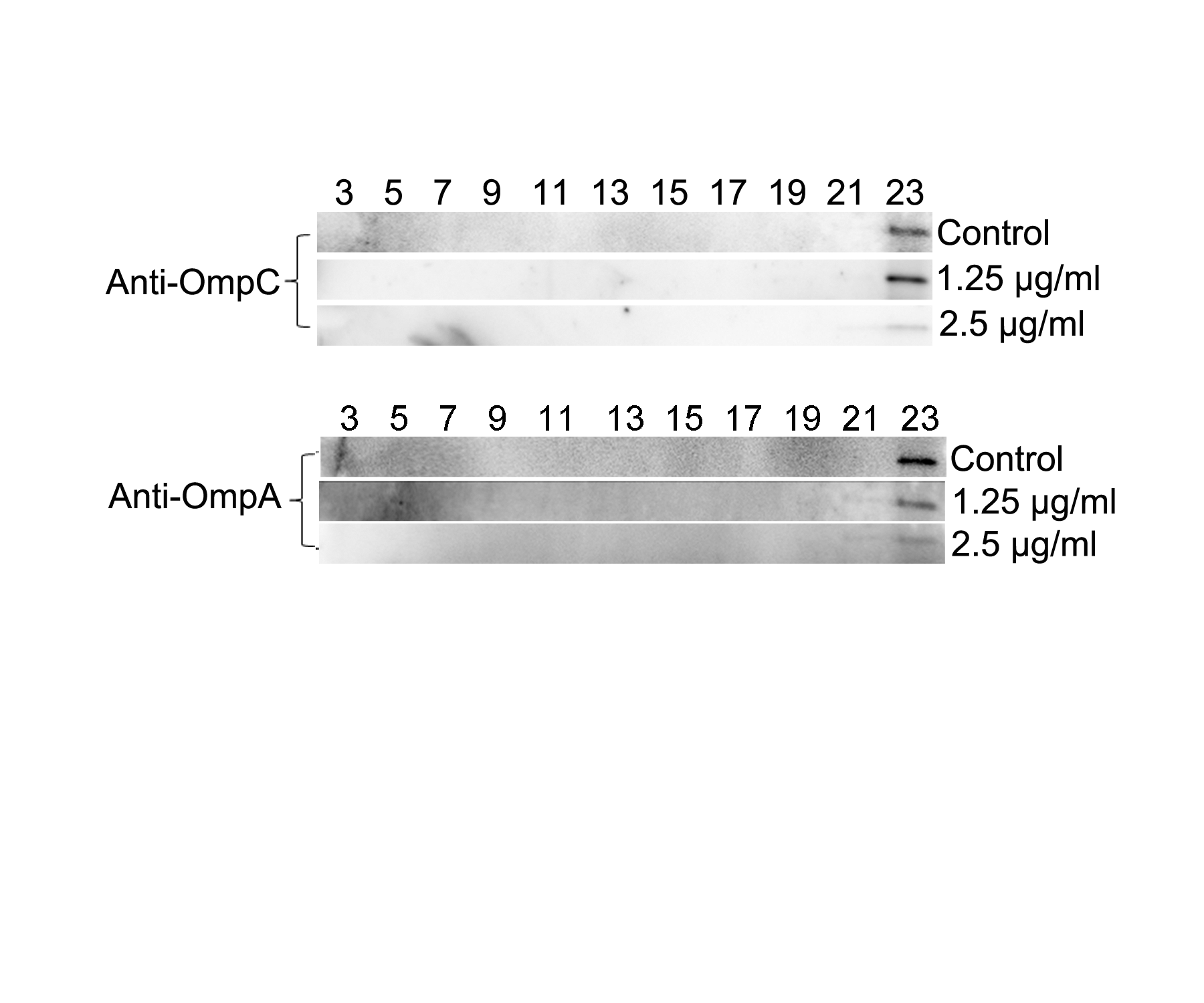

Supplement: FIGURE S2 — Western blotting bands of the outer membrane fraction. [file Image_2.TIF]

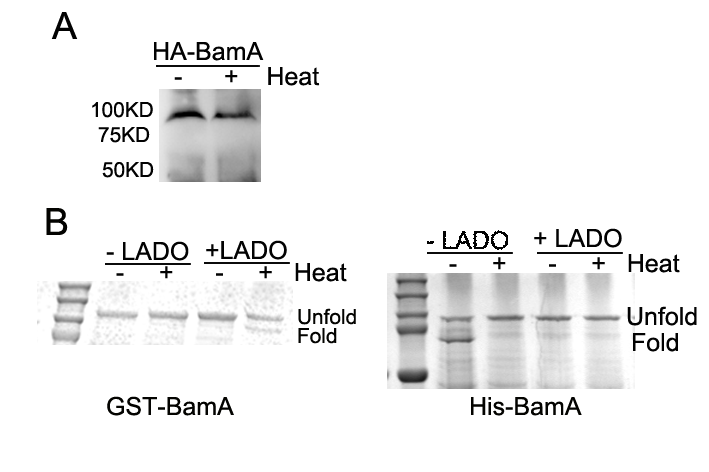

Supplement: FIGURE S3 — Analyze the folding of purified BamA and that expressed in Y2H system. (A) BamA expressed in unfolded form in Y2H system. AH109 (pAD-BamA + pBD-BamD) cultures were harvest and disrupt. The supernatant was heated or unheated in SDS sample buffer. The HA-BamA protein was determined using western blotting with anti-HA monoclonal antibodies. (B) The purified BamA protein was unfolded. SDS sample loading buffer was added to the purified BamA protein, and then heated or unheated. Proteins were separated by 10% SDS-PAGE and stained by Coomassie Blue. [file Image_3.TIF]
