# Supplementary material for: Identification of a Compound That Inhibits the Growth of Gram-Negative Bacteria by Blocking BamA–BamD Interaction
Source: Front Microbiol. 2020 Jun 19;11:1252. doi: 10.3389/fmicb.2020.01252 (PMC7316895; doi:10.3389/fmicb.2020.01252)
Supplement: TABLE S1 — The primer pairs. [file Data_Sheet_1.docx]

Table S1. The primer pairs

|  | Forward primer | Reverse primer |
| --- | --- | --- |
| *BamA* | GAACGCATAATACATATGGCGATGA | CTTTGTGGAGAAGGATCCCCAGGTT |
| *GST-BamA* | TACGCTGGATCCGCGATGAAAAAGT | CTCGATGAATTCCCAGGTTTTACCG |
| *His-BamA* | AAGGAGATATACATATGGCGATGAA | GCTCGAATTCGGATCCCGCCAGGTTTT |
| *BamD* | GAAAGTCAAAACCATATGACGCGCA | TGTTTCAGGTTTGGATCCTGTATTG |
| *His-BamD* | AAGGAGATATACATATGACGCGCAT | GCTCGAATTCGGATCCCGTGTATTGCT |

Table S2. The buffer used for protein purification

|  | **GST-BamA** | **His-BamA** | **His-BamD** |
| --- | --- | --- | --- |
| **Lysis buffer** | 2 M Urea, 50 mM Tris, 300 mM NaCl, 0.2 mM PMSF,0.1% Triton X-100, 0.5 mM EDTA, 1 mM DTT, pH 8.0 | 8 M Urea, 50 mM Tris, 300 mM NaCl, 0.1% TritonX-100, pH 8.0 | 50 mM Tris, 300 mM NaCl, 0.2 mM PMSF, 0.1% TritonX-100, pH 8.0 |
| **Binding buffer** | 50 mM Tris, 300 mM NaCl, pH 8.0 | 8 M Urea, 50 mM Tris, 300 mM NaCl, pH 8.0 | 50 mM Tris, 300 mM NaCl, pH 8.0 |
| **Washing buffer** | / | 8 M Urea, 50 mM Tris, 300 mM NaCl, 20/50 mM Imidazole, pH 8.0 | 50 mM Tris, 300 mM NaCl, 20/50 mM Imidazole, pH 8.0 |
| **Elution buffer** | 50 mM Tris, 300 mM NaCl, 20 mM GSH,  pH 8.0 | 8 M Urea, 50 mM Tris, 300 mM NaCl, 500 mM Imidazole, pH 8.0 | 50 mM Tris, 300 mM NaCl, 500 mM Imidazole, pH 8.0 |
